# *Helicobacter pylori* and its relationship with variations of gut microbiota in asymptomatic children between 6 and 12 years

**DOI:** 10.1186/s13104-018-3565-5

**Published:** 2018-07-13

**Authors:** Araceli Benavides-Ward, Fernando Vasquez-Achaya, Wilmer Silva-Caso, Miguel Angel Aguilar-Luis, Fernando Mazulis, Numan Urteaga, Juana del Valle-Mendoza

**Affiliations:** 1 School of Nutrition, Faculty of Health Sciences, Universidad Peruana de Ciencias Aplicadas, Lima, Peru; 2 School of Medicine, Research and Innovation Centre of the Faculty of Health Sciences, Universidad Peruana de Ciencias Aplicadas, Lima, Peru; 3 Instituto de Investigación Nutricional, Lima, Peru; 4 Instituto de Investigación de Enfermedades Infecciosas, Lima, Peru; 5 Puesto de Salud Callancas, Dirección Regional de Salud Cajamarca, Cajamarca, Peru

**Keywords:** *Helicobacter pylori*, Gut microbiota, School children, Peru

## Abstract

**Objective:**

To determine the variations in the composition of the intestinal microbiota in asymptomatic children infected with *Helicobacter pylori* in comparison with children without the infection.

**Results:**

Children infected with *H. pylori* doubled their probability of presenting 3 of 9 genera of bacteria from the gut microbiota, including: *Proteobacteria* (p = 0.008), *Clostridium* (p = 0.040), *Firmicutes* (p = 0.001) and *Prevotella* (p = 0.006) in comparison to patients without the infection. We performed a nutritional assessment and found that growth stunting was statistically significantly higher in patients infected with *H. pylori* (p = 0.046).

**Electronic supplementary material:**

The online version of this article (10.1186/s13104-018-3565-5) contains supplementary material, which is available to authorized users.

## Introduction

*Helicobacter pylori* (*H. pylori*) is a gram-negative bacterium, detected in up to 90% of the population of underdeveloped countries [1, 2]. However, only 15% of all infected patients develop gastrointestinal symptoms or complications, such as chronic gastritis, peptic ulcers, stomach cancer, among others [3–5]. *H. pylori* infections of the pediatric population have also been associated specific complications such as antral gastritis, failure to thrive and iron deficiency anemia [6–8].

In Peru, a high prevalence of *H. pylori* has been reported, affecting up to 80% of patients with low socioeconomic status [1]. Although the epidemiology of *H. pylori* infections among the Peruvian pediatric population is lacking, two studies in 2006 and 2011 reported a prevalence between 45.9 and 68.8% in children with gastrointestinal symptoms [9, 10].

The intestinal microbiota is composed mainly of Gram-negative anaerobic bacilli that live in the colon microenvironment with *Bacteroides* and *Fusobacterium* as the predominant genus [11]. However, due to the complex dynamics between these microorganisms and their potential roles in nutrition, metabolism and, immune protection of humans, where, despite the fact that immune responses against *H. pylori* are mixed with the involvement of T helper cells, cytotoxic T cells and NK cells, the strategies of immune evasion allow the persistence of *H. pylori* throughout life and the development of pro and anti-inflammatory immune responses [12]. There is a growing interest for researching the potential interactions between the intestinal microbiota and other important pathogens such as *H. pylori* [13].

Dysbiosis is a term for a microbial imbalance or change in the regular pattern of colonizing organisms inside the body, and it can be a consequence of numerous factors such as frequent use of antibiotics, laxatives, high-fat or low-fiber diets among others [13–16]. Furthermore, recent studies have suggested that *H. pylori* infections are significantly associated with negative interactions with the gut microbiota. It has been described that infection with *H. pylori* affects the stomach, duodenal and oral microbiota, influences the composition of the oral bacterial community and/or viceversa [17]. Another study in Germany, also reported a decrease in anaerobic bacteria, Enterobacteria, *Veillonella*, and *Clostridium* from the colon in 51 patients with dyspepsia and a confirmed *H. pylori* infection [18]. In relation to the gastric microbiota patients with negative *H. pylori* present a greater relative abundance of gammaproteobacteria, betaproteobacteria, bacteroidia and clostridial classes, as well as a greater bacterial richness and diversity, while the prodomin epsiloproteobacteria in pediatric patients with *H. pylori* infection [19] Finally, a control–case study compared patients with and without an *H. pylori* infection and demonstrated a reduction of *Clostridium* and anaerobe enteric colonies in patients that were not infected [20].

This study objective was to describe and compare the composition of the gut microbiota between asymptomatic children infected with *Helicobacter pylori*-infected vs children without the infection.

## Main text

### Methods

#### Patients and sampling

Secondary data analysis was performed from a cross-sectional study on 56 schoolchildren age between 6 and 12 years old, from San Pablo, Cajamarca. 28 fecal samples from children with *H. pylori* and 28 samples with a negative result for *H. pylori* were included for comparison.

#### Inclusion and exclusion criteria

Children who fulfilled the following criteria were included: patients must be of school age, with a positive sample for *H. pylori*, with residency within the area of study at least 6 months prior and a signed informed consent by their respective guardian.

The exclusion criteria included children who had initiated antibiotic treatment for *H. pylori*, children with ongoing antiparasitic therapy or had ingested laxatives 15 days before enrollment.

#### Ethics statement

This study has been approved by two independent Ethics Committees from *Hospital Regional de Cajamarca* and *Universidad Peruana de Ciencias Aplicadas*. Parents and caregivers signed a written consent on the previous study, which included a section to give the investigators permission to reproduce further investigations from the patients’ samples.

#### Sample preparation

One sample of feces was obtained from each participant, they were collected in sterile containers and stored at − 80 °C until processing.

#### DNA extraction

DNA was extracted from above mentioned sample aliquots using a commercial kit QIAamp DNA mini kit (Qiagen, Mississauga, Ontario) according to the manufacturer instructions. DNA extraction was subjected to a dilution of 100 µl nuclease free buffer, the DNA was analyzed immediately or stored at − 80 °C until use.

#### PCR amplification for detection *H. pylori*

Presence of *H. pylori* was determined by PCR amplification of the *23S rRNA* gene using the primers and conditions previously described [21]. The amplified products were electrophoretically analyzed, recovered and sequenced to confirm the PCR results.

#### PCR amplification for detection gut microbiota

The 13 pathogens evaluated in gut microbiota were amplified using DNA extracted of fecal samples and primers used were previously described [22]. Amplifications consisted of initial incubation at 95 °C for 2 min, followed by 40 cycles of 95 °C for 30 s; 58 °C for 30 s, and 72 °C for 30 s; with a final extension at 72 °C for 5 min. Amplified products were gel recovered, purified using SpinPrep™ Gel DNA Kit (EMD Biosciences, Madison, WI, USA) and sent to be sequenced (Macrogen Inc., Geumcheon-gu, Seoul, Korea).

#### Statistical analysis

Qualitative variables were reported as frequencies and percentages. To evaluate the difference between groups for continuous variables, a t-test or Kruskal–Wallis were applied. For categorical variables, a χ^2^ test and Fisher’s exact test were used to evaluate the difference. Probability values of p < 0.05 were considered significant. The analysis was performed using the Stata version 14.0 (Stata Corp. Texas, USA).

### Results

A total of 56 children from the San Pablo region, Cajamarca, were included in the study. Children were between 6 and 12 years old with an average of 8.9 (+2.2) years, males (51.8%) were slightly more common than females (48.2%) female (Table 1).

**Table 1:** Characteristics and habits of studied population in relation to genre of bacteria found in intestinal microbiota

	No of bacteria				Total		*p*-value
	0–2		3–9				
Gender (n/%)							
Male	14	48.3	15	55.6	29	51.8	0.391
Female	15	51.7	12	44.4	27	48.2	
Age (years)							
Age (Me/SD)	9	2.4	8.7	2.0	8.9	2.2	0.667
Hand washing before eating (n/%)							
Sometimes	5	17.2	13	48.1	18	32.1	0.014
Always	24	82.8	14	51.9	38	67.9	
Hand washing after toilet use (n/%)							
Never	1	3.4	0	0.0	1	1.8	0.018
Sometimes	2	6.9	9	33.3	11	19.6	
Always	26	89.7	18	66.7	44	78.6	
Livestock (n/%)							
Yes	25	86.2	23	85.2	48	85.7	0.563
No	4	13.8	3	11.1	7	12.5	
Access to boiled water (n/%)						0.0	
Never	4	13.8	5	18.5	9	16.1	0.337
Sometimes	13	44.8	16	59.3	29	51.8	
Always	12	41.4	6	22.2	18	32.1	
Ditch water ingestion (n/%)							
Never	24	82.8	25	92.6	49	87.5	0.242
Sometimes	5	17.2	2	7.4	7	12.5	
Diarrhea within past 3 months (n/%)							
Yes	23	79.3	18	66.7	41	73.2	0.222
No	6	20.7	9	33.3	15	26.8	
Fruit and vegetable consumption (n/%)							
Always	8	27.6	0	0.0	7	12.5	0.010
Sometimes	11	37.9	14	51.9	23	41.1	
Never	10	34.5	13	48.1	26	46.4	
Hay chewing (n/%)							
Yes	2	6.9	6	22.2	8	14.3	0.104
No	27	93.1	21	77.8	48	85.7	
Caregiver occupation (n/%)							
Agricultural	25	86.2	17	63.0	42	75.0	0.067
Other	4	13.8	9	33.3	13	23.2	
Presence of parasites (n/%)							
Yes	4	13.8	1	3.7	5	8.9	0.147
No	18	62.1	23	85.2	41	73.2	

*p* value: Statistical χ^2^ and Exact Fisher Test

Additionally, children who always consume fruits and vegetables have a lower number of bacteria in the gut microbiota (0–2 bacteria 100% vs. 3–9 bacteria 0% p = 0.010). However, those who occasionally consume fruits and vegetables or those who didn’t were more likely to have 3 or more positive bacteria in the microbiota. In addition, a marginally significant difference was found between the father’s occupation as a farmer and the number of bacteria in the children’s microbiota (p = 0.067), where 60% of sons of a farmer had 0–2 bacteria (60%) (Table 1).

We found that children with a positive sample for *H. pylori* have on average a significantly higher number of intestinal bacteria from the gut microbiota than children without an *H. pylori* infection (p = 0.033). Children with *H. pylori* had a median of 4 genera of bacteria (within a range of 0–7), while the group of children without *H. pylori* had a median of 0 genera of bacteria (within a range of 0–4). Furthermore, the number of positive bacteria from the microbiota were categorized into two groups (0–2 genera of bacteria and 3–9 genera of bacteria). In children with *H. pylori*, it was statistically more common to find 3–9 bacteria in the intestinal microbiota compared to children without the infection (p = 0.016) (Table 2).

**Table 2:** Comparison between intestinal microbiota and the presence of *H. pylori* in the school-age patients from a community in San Pablo, Cajamarca, Peru

	Total		PCR results for *H. pylori*				*p*-value
			Positive		Negative		
Presence of intestinal microbiota (n/%)							
Yes	32	57.1	20	71.43	12	42.86	0.029
No	24	42.9	8	28.57	16	57.14	
Mean N of bacteria (mean/RI)							
Bacterial microbiota	2	0–6	4	0–7	0	0–4	0.033
No of bacteria (n/%)							
0–2 bacteria	29	51.8	10	35.71	19	67.86	0.016
3–9 bacteria	27	48.2	18	64.29	9	32.14	
Identified bacteria (n/%)							
*Actinobacteria*	3	5.4	3	10.7	0	0.0	0.118
*Bacteroides*	7	12.5	0	0.0	7	25.0	0.005
*Bacteroidetes*	14	25.0	10	35.7	4	14.3	0.061
*Bifidobacterium*	10	17.9	6	21.4	4	14.3	0.364
*Clostridium*	17	30.4	12	42.9	5	17.9	0.040
*Enterococcus*	6	10.7	5	17.9	1	3.6	0.096
*Eubacterium*	17	30.4	10	35.7	7	25.0	0.281
*Firmicutes*	20	35.7	16	57.1	4	14.3	0.001
*Fusobacterium*	1	1.8	0	0.0	1	3.6	0.500
*Lactobacillus*	21	37.5	13	46.4	8	28.6	0.135
*Prevotella*	22	39.3	16	57.1	6	21.4	0.006
*Proteobacteria*	30	53.6	20	71.4	10	35.7	0.008
*Veillonella*	2	3.6	0	0.0	2	7.1	0.245

*p*-value: Statistical χ^2^ and Exact Fisher test

*Med/RI* Mediana rango interquartile

**Fig. 1:**
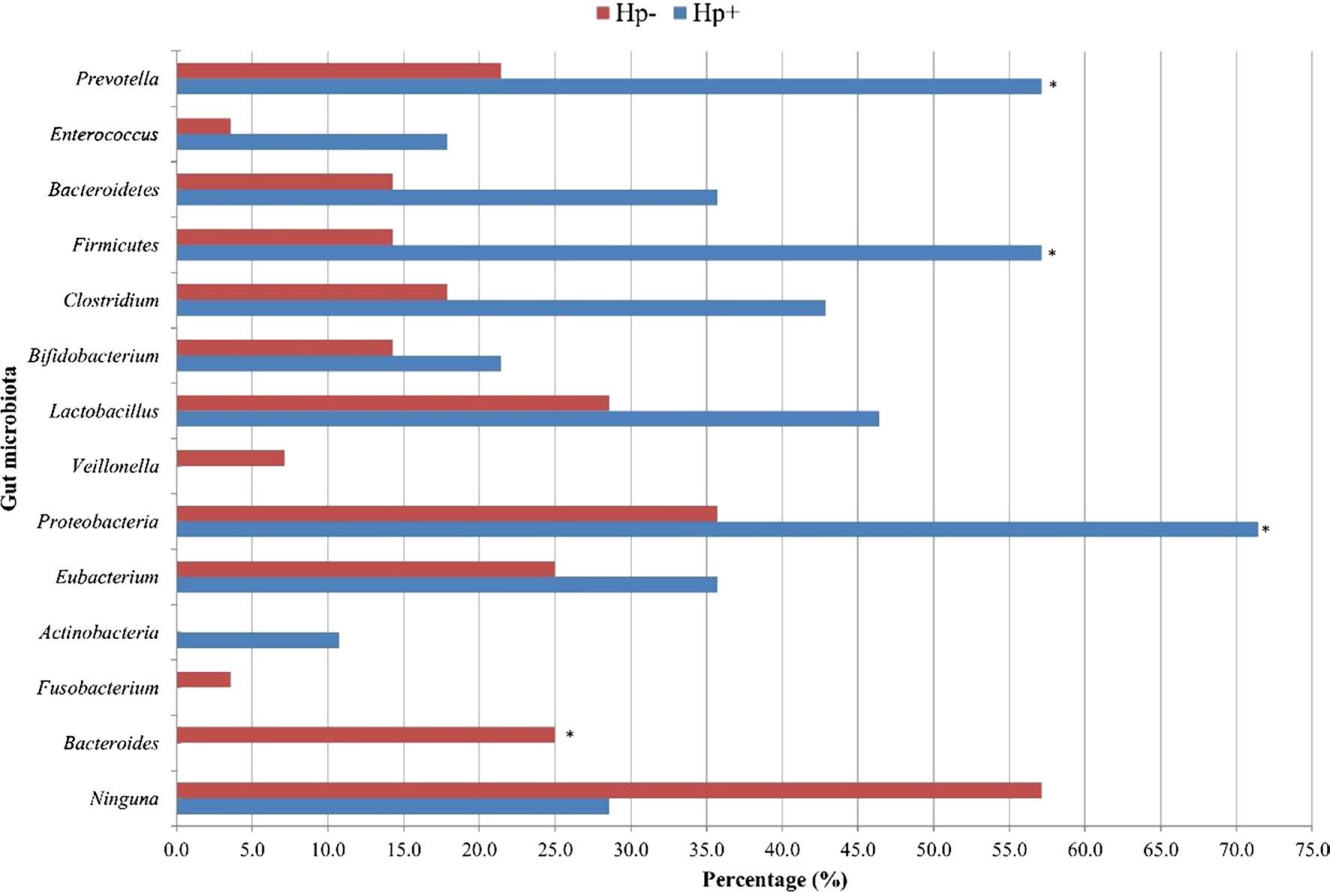
Difference in composition of 13 genera of bacteria from the intestinal microbiota of school-age children (*H. pylori *+ vs. *H. pylori −*)

In children with *H. pylori* the presence of *Proteobacteria* (p = 0.008), *Clostridium* (p = 0.040), *Firmicutes* (p = 0.001) and *Prevotella* (p = 0.006) was significantly higher. On the contrary, *Bacteroides* (p = 0.029) was more common in patients without *H. pylori* (Fig. 1).

A nutritional assessment was performed in our study population using the Z-scores system. No children with malnutrition were observed, 80.4% of children have an adequate weight, 17.9% were overweight, and only one child was obese. However, more than half of children (61.1%) showed growth stunting, and this was significantly higher among the children with *H. pylori* (74.1% vs. 48.2%, p = 0.046). The average hemoglobin level was 13.1 g/dl, and only one child had mild anemia (Additional file 1: Table S1).

### Discussion

In our study, we have observed that the presence of *H. pylori* may have a significant impact on the gut microbiota composition of school children. In our population, children with *H. pylori* had twice chances of having an increased number and variety of bacteria from their colonic microbiota. Similar results have been reported in a Chilean study, were a more diverse microbiota was described in children with *H. pylori* chronic infections [23]. This increased diversity could be related to changes in the gastric pH, the role of the microbiota as an immune barrier, drinking untreated water, among others [13, 24, 25].

The intestinal microbiota serves as an immune barrier that competes for nutrients and space against pathogenic bacteria [25]. We observed a higher number of beneficial bacteria such as *Bacteroidetes*, *Lactobacillus*, and *Bifidobacterium* in children with *H. pylori*, but this difference was not significant. However, similar results have been reported by Buhling et al. [18] in a study were lactobacilli more commonly observed in patients infected with *H. pylori*. The presence of these bacteria could be beneficial since *Bifidobacterium* secrete metabolites that stimulate the epithelial receptors, enhancing the immune function of the whole body [26]. Higher levels of bifidobacteria help the maturation of the intestinal lining and together with lactobacilli maintain their integrity, regulate the pH of the body, serve as antibiotics, antivirals and even natural antifungals that regulate immunity and control inflammation [13]. Moreover, Lactobacillus is characterized by generating an acidic environment and thus reduce the growth of potentially harmful bacteria.

Other results reported in the study by Buhling et al., such as the decrease in the levels of *Enterobacteria* and *Clostridium* in patients with *H. pylori* are contrary to our results. However, these differences may be due to the study was done in adults from Germany, where hygiene conditions are very different from rural areas from Peru [18].

Several studies have shown a direct correlation between *H. pylori* infection and failure to thrive, especially in patients from developing countries. This association may be due to hypochlorhydria generated by *H. pylori* infection, which interferes with nutrients absorption and increases susceptibility to enteric infections [27]. Additionally, subjects infected with *H. pylori* commonly have lower levels of ghrelin and a higher concentration of leptin producing an anorectic effect that eventually leads to malnutrition and failure to thrive [7]. Consequently, we found an association between the short stature and the presence of *H. pylori* in our study population.

*Helicobacter pylori* infection has been associated with iron deficiency anemia, due to blood loss from gastroduodenal lesions, gastric atrophy hypochlorhydria, and competition for nutrient between the bacteria and the host [8]. However, in the present study, no significant difference was found between the two groups studied probably because only one child had mild anemia. In addition, hemorrhagic lesions and severe gastric atrophy are uncommon in the pediatric population, as they usually develop during a long-term infection [28]. For example, a multicenter study that evaluated 1233 children with dyspeptic symptoms and confirmed *H. pylori* infection, found that less than 5% of children under the age of 12 had an associated peptic ulcer [29]. Therefore, despite children being an asymptomatic population, the prevalence of peptic ulcer disease due to *H. pylori* is low, which leads us to believe that in our study, the prevalence would be even lower.

A high intake of fruits and vegetable consumption has been reported to have many benefits on the maintenance of the intestinal microbiota due to many factors including the high intake of fiber. However, in this study, our findings show the opposite, where children who always consume fruits and vegetables had a lower number of bacteria in the gut microbiota. This discrepancy may be due to the low consumption per capita of fruits and vegetables in Peru [30]. As we observed in our study, less than 15% of children reported daily consumption of fruits and vegetables. On the other hand, it was not possible to evaluate dietary habits related to infection with *H. pylori*, such as the consumption of salt or cured meats because the corresponding information was not available.

This study is the first to compare 13 genera of intestinal microbiota among Peruvian children from a rural community with a confirmed *H. pylori* infection. However, these bacteria were chosen because they are representative of the maintenance of the normal intestinal flora and it has been described that the absence or imbalance of some of them may be closely related to the development of gastrointestinal diseases. Our study determined that children infected with *H. pylori* increased numbers of bacteria from the gut microbiota, including *Proteobacteria*, *Clostridium*, *Firmicutes* and *Prevotella* in comparison to patients without the infection. However, the information available regarding the interactions between *H. pylori* and the intestinal bacteria from the microbiota is still limited.

### Limitations

A limitation was the small number of patients we could include for analysis. The study design does not allow us to identify the temporal sequence, thus we cannot conclude if the *H. pylori* had a direct impact on the microbiota, or if any dysbiosis in the intestinal bacterial flora may have favored an *H. pylori* infection. For this reason, a longitudinal study is recommended.

## Additional file

**Additional file 1: Table S1.** Nutritional status assessment in relation to the presence of *H. pylori* and the number of bacterial genre present in the intestinal microbiota.

## Abbreviations

PCR: polymerase chain reaction
DNA: deoxyribonucleic acid
rRNA: ribosomal ribonucleic acid
bp: base pairs
