# Supplementary material for: Helicobacter pylori and its relationship with variations of gut microbiota in asymptomatic children between 6 and 12 years
Source: BMC Res Notes. 2018 Jul 13;11:468. doi: 10.1186/s13104-018-3565-5 (PMC6043948; doi:10.1186/s13104-018-3565-5)
Supplement: Supplementary file 1 — Additional file 1: Table S1. Nutritional status assessment in relation to the presence of H. pylori and the number of bacterial genre present in the intestinal microbiota. [file 13104_2018_3565_MOESM1_ESM.docx]

Table S1**.** **Nutritional status assessment in relation to the presence of *H. pylori* and the number of bacterial genre present in the intestinal microbiota.**

|  | **PCR results for *H. pylori*** | | | |  |  |  | **N° of bacteria** | | | |  |  |  |
| --- | --- | --- | --- | --- | --- | --- | --- | --- | --- | --- | --- | --- | --- | --- |
| **Variable** | **Negative** | | **Positive** | | **Total** | | **p** | **0-2** | | **3-9** | | **Total** | | **p** |
|  | **n** | **%** | **n** | **%** | **n** | **%** |  | **n** | **%** | **n** | **%** | **n** | **%** |  |
| **BMI/A Z-Score (Median/SD)** | **0.3** | **0.6** | **0.5** | **0.8** | **0.4** | **0.7** | **0.199** | **0.4** | **0.7** | **0.4** | **0.6** | **0.4** | **0.6** | **0.948** |
| Normal | 23 | 82.1 | 22 | 78.6 | 45 | 80.4 | 1.000 | 22 | 48.9 | 23 | 51.1 | 45 | 100.0 | 0.731 |
| Overweight | 5 | 17.9 | 5 | 17.9 | 10 | 17.9 |  | 6 | 60.0 | 4 | 40.0 | 10 | 100.0 |  |
| Obesity | 0 | 0.0 | 1 | 3.6 | 1 | 1.8 |  | 1 | 100.0 | 0 | 0.0 | 1 | 100.0 |  |
| **Z-score H/A (Median/SD)** | **-2.1** | **0.9** | **-2.4** | **0.8** | **-2.2** | **0.9** | **0.168** | **-2.3** | **1.1** | **-2.4** | **0.9** | **-2.3** | **1.0** | **0.744** |
| Normal Height | 14 | 51.9 | 7 | 25.9 | 21 | 38.9 | 0.046 | 14 | 66.7 | 7 | 33.3 | 21 | 100.0 | 0.046 |
| Stunted Growth | 13 | 48.2 | 20 | 74.1 | 22 | 61.1 |  | 13 | 39.4 | 20 | 60.6 | 22 | 100.0 |  |
| **Hemoglobin** | **13.1** | **0.8** | **13.1** | **0.9** | **13.1** | **0.8** | **0.918** | **13.2** | **0.8** | **13.1** | **0.9** | **13.1** | **0.8** | **0.678** |
| Mild Anemia | 1 | 18.5 | 0 | 0.0 | 1 | 1.8 | 0.879 | 1 | 100.0 | 0 | 0.0 | 1 | 100.0 | 0.527 |
| Without Anemia | 26 | 96.3 | 28 | 100.0 | 54 | 98.2 |  | 28 | 51.8 | 26 | 48.2 | 54 | 100.0 |  |

BMI/E: Body mass index for age, H/E: Height for age, SD: Standard deviation.

*p* value: Statistical T student, Chi2 and Exact Fisher test.
